# Reason of Discontinuation After Transarterial Chemoembolization Influences Survival in Patients with Hepatocellular Carcinoma

**DOI:** 10.1007/s00270-018-2118-6

**Published:** 2018-11-28

**Authors:** Tim A. Labeur, R. Bart Takkenberg, Heinz-Josef Klümpen, Otto M. van Delden

**Affiliations:** 1 Cancer Center Amsterdam, Amsterdam, The Netherlands; 2 Department of Medical Oncology, Amsterdam University Medical Centers, University of Amsterdam, Meibergdreef 9, Amsterdam, The Netherlands; 3 Department of Gastroenterology and Hepatology, Amsterdam University Medical Centers, University of Amsterdam, Meibergdreef 9, Amsterdam, The Netherlands; 4 Department of Radiology and Nuclear Medicine, Amsterdam University Medical Centers, University of Amsterdam, Meibergdreef 9, 1105 AZ Amsterdam, The Netherlands

**Keywords:** Hepatocellular carcinoma, Transarterial chemoembolization, TACE, Discontinuation, Time to untreatable progression by chemoembolization, Survival

## Abstract

**Background:**

Transarterial chemoembolization (TACE) for intermediate-stage hepatocellular carcinoma (HCC) is often repeated until unTACEable progression (UTP) occurs. There is little data on the various reasons for stopping TACE and its consequences for subsequent treatment and survival.

**Aim:**

To assess the impact of the various reasons of UTP on survival and consequences for subsequent treatments.

**Methods:**

Consecutive HCC patients who underwent TACE between 2003 and 2016 were analyzed retrospectively for the reason of TACE discontinuation. UTP was defined according to the EASL guidelines, considering radiological pattern of progression, liver function and performance status (PS). Overall and post-progression survival (OS, PPS) for different reasons of TACE discontinuation were compared. The correlation between time to untreatable progression by chemoembolization (TTUPc) and OS was analyzed.

**Results:**

One hundred and sixty-six patients (BCLC-A 40%, BCLC-B 54%, BCLC-C: 7%) were included, undergoing a median of 2 TACE procedures with a median OS of 22.1 months (95% CI 17.4–26.7). UTP occurred in 116 patients (70%) after a median TTUPc of 11.6 months (95% CI 7.8–15.4). There was a strong positive correlation (*ρ* = 0.816, *p* < 0.001) between TTUPc and OS. The main reason of UTP was radiological progression (61%), which was mostly intrahepatic (75%). Hepatic decompensation and worsening of PS were independent predictors of OS and PPS.

**Conclusion:**

The majority of HCC patients treated with TACE have UTP due to intrahepatic tumor progression with preserved liver function and PS, making them potential candidates for subsequent liver-directed or systemic treatment. TTUPc may be a valuable surrogate endpoint for OS in patients treated with TACE.

**Level of Evidence:**

Level II, prognosis study.

**Electronic supplementary material:**

The online version of this article (10.1007/s00270-018-2118-6) contains supplementary material, which is available to authorized users.

## Introduction

Transarterial chemoembolization (TACE) currently is the cornerstone of treatment for patients with intermediate-stage hepatocellular carcinoma (HCC) [1, 2], based on two randomized controlled trials showing survival benefit of TACE compared with best supportive care (BSC) in patients with unresectable HCC [3, 4]. The beneficial effect of TACE was confirmed by two meta-analyses [5, 6]. However, with the expanding number of loco-regional (radioembolization) and systemic treatments (sorafenib [7], lenvatinib [8], regorafenib [9], cabozantinib [10], ramucirumab [11] and nivolumab [12]) for HCC, the guideline-endorsed concept of timely switch to alternative treatments [1], or treatment stage migration, is becoming increasingly relevant.

TACE can be performed repeatedly, but the potential survival benefit of each TACE should be carefully weighed against the risk of damaging normal hepatocytes and worsening liver function which may preclude subsequent treatments and potentially impair survival outcomes [13]. This has led to several scoring systems designed to identify the best candidates for TACE in order to maximize treatment benefit and to select candidates for alternatives therapies [14]. Current guidelines have not yet endorsed these predictive models, but underscore the importance of switching to alternative treatments in case of ‘unTACEable progression’ (UTP) [1]. The definition of UTP is based on expert opinion and varies between centers and countries [15–17]. Most definitions include radiological progression and deterioration of liver function or performance status. There is limited data on the impact of the various reasons for UTP in clinical practice, although this strongly influences the choice for a next-line treatment. The selection of a subsequent treatment is often based on the radiological pattern of tumor progression after TACE, i.e., progression of intrahepatic lesions, appearance of macrovascular invasion (MVI) or extrahepatic spread (EHS). Radiological pattern of progression has been shown to impact post-progression survival of HCC patients treated with sorafenib [18]. Prior studies have reported the incidence of tumor progression [3, 4] and pattern of tumor recurrence following TACE [19–21], but data on the prognostic impact of pattern of tumor progression following TACE are lacking. Lastly, the advent of multiple lines of subsequent treatments is making it increasingly difficult to assess the benefit of TACE based on overall survival (OS) alone. Novel surrogate outcomes such as time to untreatable progression by chemoembolization (TTUPc) [15, 22] have been proposed as a useful parameter for treatment guidance and valuable endpoint of future trials involving TACE. However, TTUPc requires validation in clinical practice.

This retrospective study of patients with HCC treated with TACE aims to (1) analyze the reason of UTP and radiological pattern of tumor progression and assess the impact on subsequent treatments and survival outcomes, (2) determine whether TTUPc is a useful surrogate parameter for estimating TACE benefit in terms of OS.

## Methods

### Study Population

This retrospective study was approved by the Institutional Review Board, and the need for informed consent was waived (reference number W17_420#17.488).

From February 2003 to November 2016, consecutive patients with liver-confined HCC and preserved liver function (Child–Pugh ≤ B7) who underwent TACE at our tertiary referral hospital were included. Patients were identified by querying the electronic patient registration systems and the institutional radiology archive.

### Diagnostic Workup and Treatment Algorithm

HCC was diagnosed by pathology or imaging criteria according to the European Association for the Study of the Liver (EASL) guidelines [1]. All patients were staged with 4-phase contrast-enhanced computed tomography (CT) or magnetic resonance imaging (MRI) and discussed in the HCC multidisciplinary team (MDT). Patients were considered for TACE according to the Barcelona Clinic Liver Cancer (BCLC) algorithm [1, 23]. Accordingly, TACE was considered in patients with BCLC-B, or those with BCLC-A in whom surgery or radiofrequency ablation (RFA) was not deemed possible. Patients with portal vein invasion (BCLC-C) only were considered for TACE, if tumor invasion was limited to segmental portal veins.

TACE was performed using the standard technique as described previously [24]. Before 2008, conventional TACE (cTACE) was performed using an emulsion of doxorubicin (50–75 mg/m^2^) and lipiodol followed by gelatin sponge. Since 2008, patients underwent TACE with drug-eluting beads (DEB-TACE) loaded with doxorubicin (75–150 mg)(DC beads 100–300 µm, Terumo Europe, Belgium). Patients undergoing either cTACE or DEB-TACE were evaluated in this study as both techniques have similar survival benefit [25]. Follow-up after single- or multi-session TACE included clinical, biochemical and radiological assessment after 6 weeks and every 3 subsequent months. Radiological response was assessed by multiphasic CT or MRI using the modified response evaluation criteria in solid tumor (mRECIST) criteria [14]. All patients were re-evaluated in the MDT after each follow-up visit, and additional TACE was performed in cases of non-complete response or appearance of intrahepatic recurrence. At UTP, patients were considered for subsequent treatment including sorafenib (≥ 2008) and radioembolization (≥ 2012).

### Outcomes

According to the EASL guidelines, UTP is a clinical profile that prohibits further TACE treatment [1], defined as: radiological tumor progression, including intrahepatic growth or non-response of target lesions after ≥ 2 TACE, or occurrence of extrahepatic spread (EHS) or macrovascular invasion (MVI); hepatic decompensation (Child–Pugh ≥ B8, uncontrolled ascites or encephalopathy); or worsening of performance to Eastern Cooperative Oncology Group performance status (ECOG PS) > 2. In case of radiological tumor progression, this was further specified according to radiological pattern of progression. OS was measured from date of first TACE to date of death or last known date to be alive. Survival status was checked using the municipal records database on May 4, 2018. When TACE was used as bridging treatment to curative resection or liver transplantation, OS was censored on date of surgery. OS was divided into time to untreatable progression by chemoembolization (TTUPc) and post-progression survival (PPS) as proposed by Kudo et al. [16] (Supplementary Fig. 1). TTUPc was defined from date of first TACE to date of UTP or censored at the time of last radiological evaluation. Patients without UTP who did not have at least one radiological evaluation were excluded from TTUPc analysis. PPS was defined as the period from date of UTP to date of death or last follow-up.

The OS and PPS were compared according to different reasons of UTP. Moreover, the radiological pattern of progression was analyzed in a subgroup of patients with preserved liver function and ECOG PS, thereby eliminating the competing risk of impairment of ECOG PS and liver function.

### Statistical Analysis

Categorical variables were described as frequencies with percentages and continuous variables as medians with interquartile ranges (IQR). Time-to-event data were estimated by Kaplan–Meier method with plot and median (95% confidence interval [95% CI]). Differences in survival rate were assessed by log-rank test. To assess the association between survival (OS and PPS) and the reason of UTP and radiological pattern, these were analyzed in a multivariable Cox proportional hazards analysis, adjusting for known prognostic factors [26] and additional factors that were associated with survival in univariable analysis (*p *< 0.1). Hazard ratios (HR) with 95% confidence intervals (CI) were calculated. The relationship between TTUPc/PPS and OS was assessed with the Spearman correlation (*ρ*) test in the whole cohort. For all statistical tests, a two-tailed *p* value of < 0.05 was considered statistically significant. All analyses were performed using IBM SPSS Statistics for Windows Version 24.0 (IBM Corp., Armonk, NY, USA).

## Results

### Patient Characteristics Prior to First TACE

**Fig. 1:**
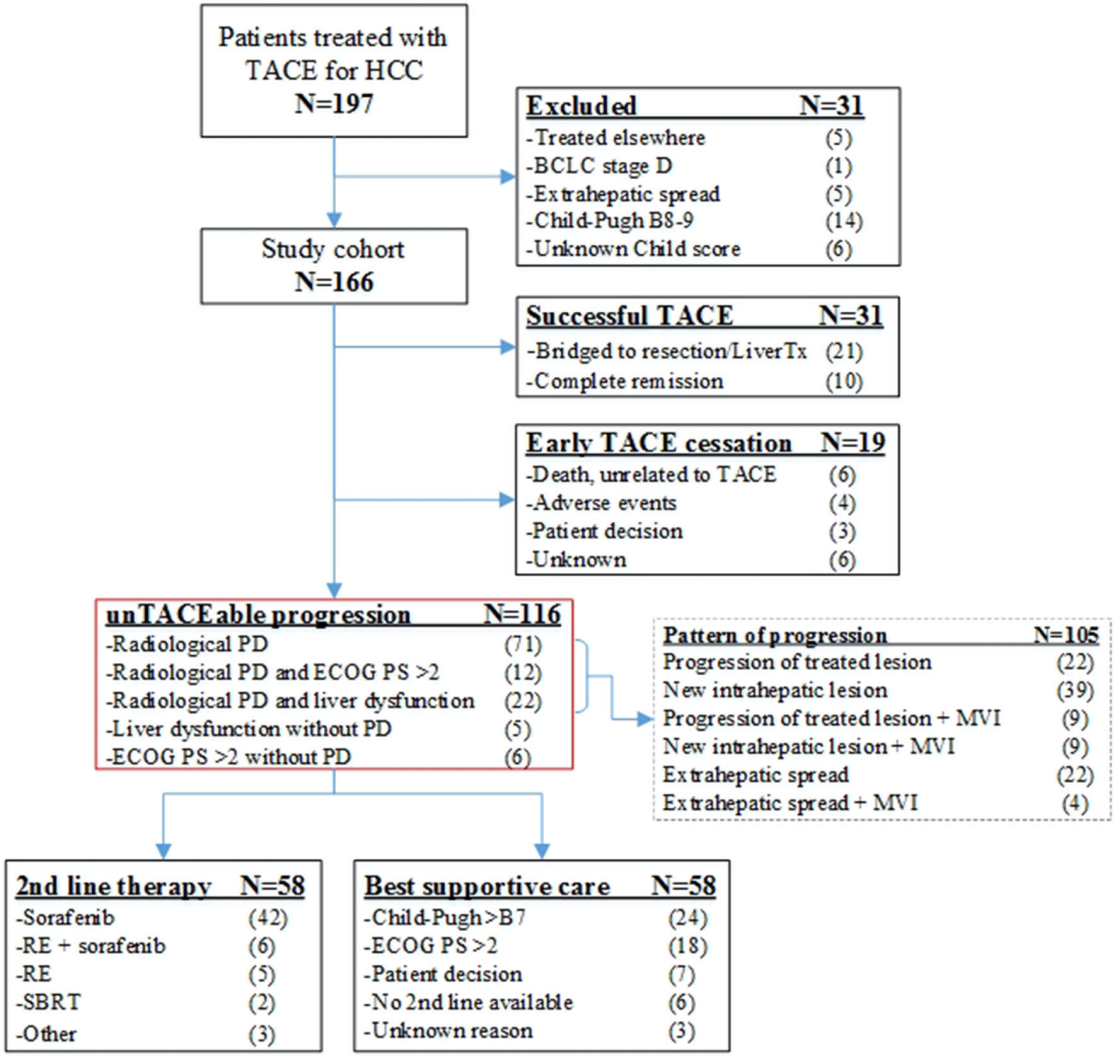
Consort flow diagram. *BCLC* Barcelona Clinic Liver Cancer; *ECOG PS* Eastern Cooperative Oncology Group performance status; *HCC* hepatocellular carcinoma; *LiverTx* liver transplantation; *MVI* macrovascular invasion; *PD* progressive disease; *RE* radioembolization; *SBRT* stereotactic body radiotherapy (SBRT); *TACE* transarterial chemoembolization

Between February 2003 and November 2016, 197 patients who underwent TACE for HCC were identified at our institution. After exclusion of 31 patients, 166 patients met the eligibility criteria and formed the study cohort (Fig. 1). The baseline demographic, clinical and imaging characteristics are summarized in Table 1. Prior to TACE, 88 (54%) patients had intermediate-stage HCC (BCLC-B) and 54 (40%) patients had early-stage HCC (BCLC-0/A) which was treated with TACE due to ineligibility for liver resection, transplantation or local ablation. Eleven (7%) patients had advanced stage (BCLC-C), due to segmental MVI.

**Table 1:** Baseline characteristics of patients (prior to first TACE)

Characteristic	All patients (*N *= 166)
Age—years (IQR)	69 (61–74)
Males—*n* (%)	129 (78)
Etiology—*n* (%)	
Alcohol	63 (38)
HBV	24 (15)
HCV	53 (32)
NAFLD/NASH	16 (10)
Other/unknown	10/19 (6/11)
Cirrhosis—*n* (%)	146 (89)
Child–Pugh class—*n* (%)	
A/B7	133/13 (91/9)
ECOG PS—*n* (%)	
0/1/2	94/58/12 (57/35/7)
BCLC stage—*n* (%)	
0 or A/B/C	67/88/11 (40/53/7)
Number of tumor nodules—*n* (%)	
1/2–3/> 3	54/68/44 (33/41/27)
Size of largest nodule—mm (IQR)	46 (34–61)
Macroscopic vascular invasion—*n* (%)	11 (7)

*BCLC* Barcelona Clinic Liver Cancer classification; *ECOG PS* Eastern Cooperative Oncology Group performance status; *HBV* hepatitis B virus; *HCV* hepatitis C virus; *IQR* interquartile range; *NAFLD*/*NASH* non-alcoholic fatty liver disease/non-alcoholic steatohepatitis; *TACE* transarterial chemoembolization

### Treatment Details and Reason of unTACEable Progression

Patients underwent a median of 2 TACE sessions (range 1–7), mostly DEB-TACE (78%). Treatment details are summarized in Supplementary Table 1. At the time of database lock, May 4, 2018, 50 patients did not have UTP, for example, due to complete remission or liver transplantation after TACE, or early TACE cessation (Figure 1). Consequently, 116 patients developed UTP. Most of these had radiological tumor progression (*n* = 105, 91%), sometimes in combination with deteriorated ECOG PS (*n* = 12, 7%) or hepatic decompensation (*n* = 22, 19%). The radiological pattern of tumor progression is specified in detail in Fig. 1, including in intrahepatic progression (*n* = 61, 58%), intrahepatic progression with MVI (*n* = 18, 17%) and EHS (*n* = 26, 25%). When considering all (*n* = 27) patients with hepatic decompensation at the time of TACE discontinuation, with (*n* = 22) or without (*n* = 5) tumor progression, only 3/27 (11%) patients recovered to Child–Pugh ≤ B7 rendering them potentially eligible for subsequent treatment.

After UTP, subsequent treatment was given in 58 patients, mainly sorafenib (*n* = 42) or radioembolization (*n* = 11). Patients who received best supportive care (BSC, *n* = 58) often had hepatic decompensation (*n* = 24) or ECOG PS > 2 (*n* = 18) prohibiting treatment. Thirteen patients did not receive subsequent treatment despite eligibility, i.e., due to patient decision or unavailability of a subsequent line of treatment (< 2008).

### Overall Survival, Time to unTACEable Progression and Post-Progression Survival

**Fig. 2:**
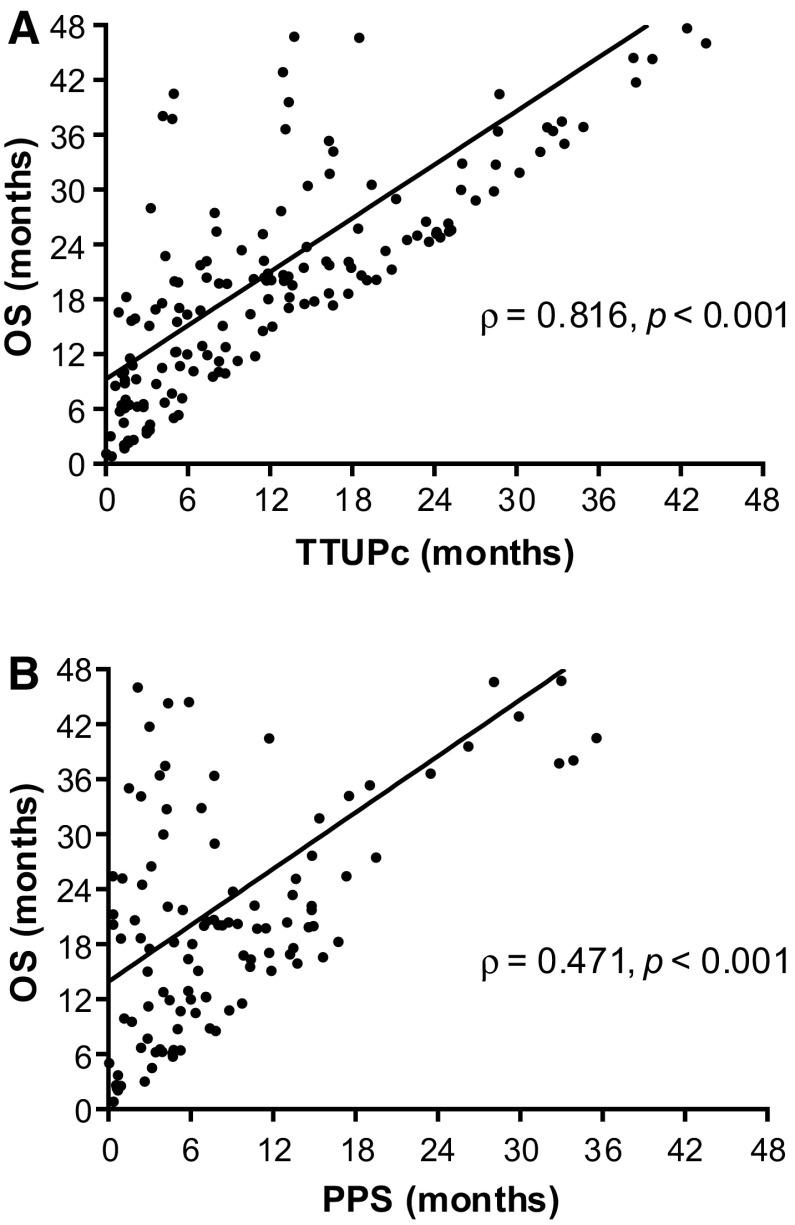
Scatter plots of the correlation between overall survival (OS) and **A** time to untreatable progression (TTUPc) **B** post-progression survival (PPS)

After a median follow-up of 40.5 months (95% CI 27.6–53.3), 115 out of 166 (69%) patients had died. The median OS was 22.1 months (95% CI 17.4–26.7). One patient did not have ≥ 1 imaging evaluation; thus, 165 patients were available for TTUPc analysis. UTP occurred in 116 out of 165 patients (70%) after a median TTUPc of 13.3 months (95% CI 10.4–16.3). The median PPS and OS in patients who had UTP (*n* = 116) was 7.1 months (95% CI 5.6–8.6) and 20.1 months (95% CI 18.4–21.8). Spearman correlation analysis showed a (very) strong positive correlation (*ρ* = 0.816, *p *< 0.001) between TTUPc and OS, and a moderate positive correlation (*ρ* = 0.471, *p *< 0.001) between PPS and OS (Fig. 2A/B).

### Impact of Reason of UTP, Pattern of Progression and Subsequent Treatment on OS and PPS

To assess the association between the various reasons of UTP and survival, patients were categorized in 3 subgroups according to the main reason of UTP:

(A) 68 patients had radiological progression with preserved liver function and performance status,
(B) 18 patients had worsening of performance status to ECOG PS > 2, and
(C) 27 patients developed hepatic decompensation.

**Fig. 3:**
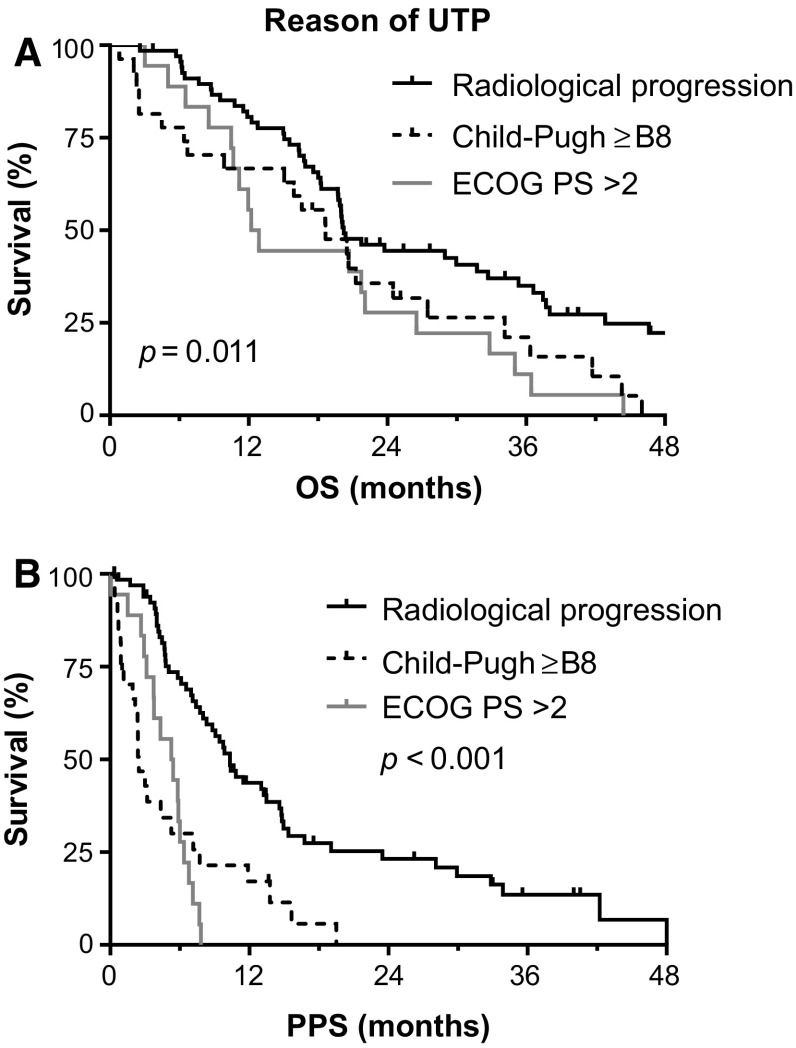
**A** Overall survival (OS) and **B** post-progression survival (PPS) according to reason of unTACEable progression (UTP). *ECOG PS* Eastern Cooperative Oncology Group performance status

Three patients had radiological progression, but data on liver function or ECOG PS were lacking. There were significant differences in OS and PPS depending on the reason of UTP (Fig. 3A, B). Patients in group A had a median OS of 20.1 months (95% CI 18.0–22.2) compared with 12.2 (95% CI 10.3–14.1) and 18.6 months (95% CI 12.4–24.9) in groups B and C, respectively (overall log-rank *p *= 0.011). In these subgroups, the median PPS was (A) 10.3 (95% CI 8.0–12.6), (B) 5.3 (95% CI 3.0–7.5) and (C) 2.4 months (95% CI 1.7–3.2), respectively (overall log-rank *p* < 0.001). After correction for known predictors, the reason of UTP remained an independent predictor of both OS and PPS in multivariable analysis (Tables 2 and 3).

**Table 2:** Multivariable Cox regression analysis for overall survival

	Whole cohort n = 166		unTACEable progression n = 116	
	HR [CI 95%]	*p* value*	HR [CI 95%]	*p* value*
*Prior to first TACE*				
ECOG PS 2 (Ref: 0–1)	2.54 (1.26–5.12)	**0.009**	1.69 (0.70–4.07)	0.242
Number of nodules (Ref: 1)	Ref	–	Ref	–
2–3	1.58 (0.91–2.73)	0.104	1.17 (0.65–2.11)	0.598
> 3/diffuse	3.04 (1.71–5.40)	**< 0.001**	2.02 (1.07–3.81)	**0.031**
Tumor size > 46 mm	1.25 (0.83–1.88)	0.282	1.40 (0.89–2.21)	0.149
Macrovascular invasion	2.56 (1.20–5.47)	**0.015**	2.44 (1.00–6.00)	0.051
Log_10_ AFP	1.51 (1.26–1.82)	**< 0.001**	1.59 (1.31–1.94)	**< 0.001**
*At unTACEable progression*				
unTACEable progression	1.88 (1.06–3.32)	**0.031**	NA (all progressors)	
Main reason (Ref: radiological PD)	–	–	Ref	–
Liver dysfunction	–	–	2.20 (1.23–4.01)	**0.008**
ECOG PS > 2	–	–	2.09 (1.10–4.00)	**0.025**

Univariable analysis shown in Supplementary Table 2

*AFP* Alpha-fetoprotein; *CI* 95 95% confidence interval; *ECOG PS* Eastern Cooperative Oncology Group performance status; *HR* hazard ratio; *NA* not applicable; *PD* progressive disease; *Ref* reference; *TACE* transarterial chemoembolization

*In bold: *p *< 0.05

**Table 3:** Univariable and multivariable Cox regression analysis for post-progression survival

	Univariable		Multivariable	
	HR [CI 95%]	*p* value*	HR [CI 95%]	*p* value**
*Prior to TACE-1*				
Female sex	1.46 (0.92–2.33)	0.108		
Age > 65	1.02 (0.68–1.53)	0.914		
HBV	1.02 (0.58–1.81)	0.935		
HCV	1.07 (0.69–1.67)	0.769		
Alcohol	1.10 (0.73–1.67)	0.641		
ECOG PS 2 (Ref: 0–1)	1.70 (0.81–3.58)	0.160		
Child–Pugh score B7 (Ref: A5–A6)	1.51 (0.73–3.14)	0.267		
BCLC stage (Ref: 0/A)	Ref	–		
B	1.32 (0.86–2.04)	0.284		
C	1.82 (0.80–4.16)	0.153		
Number of nodules (Ref: 1)	Ref	–	Ref	–
2–3	1.36 (0.82–2.26)	0.238	1.44 (0.84–2.45)	0.185
> 3/diffuse	1.62 (0.96–2.74)	**0.071**	1.21 (0.66–2.21)	0.544
Tumor size > 46 mm	0.83 (0.56–1.25)	0.372		
Macrovascular invasion	1.53 (0.71–3.32)	0.280		
Log_10_ AFP	1.30 (1.10–1.53)	**0.003**	1.36 (1.14–1.62)	**0.001**
*At unTACEable progression*				
unTACEable progression	NA (all progressors)		NA (all progressors)	
Reason (Ref: radiological PD)	Ref	–	Ref	–
Liver dysfunction	3.10 (1.88–5.11)	**< 0.001**	3.24 (1.82–5.74)	**< 0.001**
ECOG PS > 2	3.74 (2.07–6.75)	**< 0.001**	3.83 (2.09–7.01)	**< 0.001**

*In bold: included in multivariable analysis (*p* value < 0.1)

**In bold: *p* value < 0.05

*AFP* Alpha-fetoprotein; *BCLC* Barcelona Clinic Liver Cancer classification; *CI* 95 95% confidence interval; *ECOG PS* Eastern Cooperative Oncology Group performance status; *HBV* hepatitis B virus; *HCV* hepatitis C virus; HR, hazard ratio; *NA* not applicable; *PD* progressive disease; *Ref* reference; *TACE* transarterial chemoembolization

**Fig. 4:**
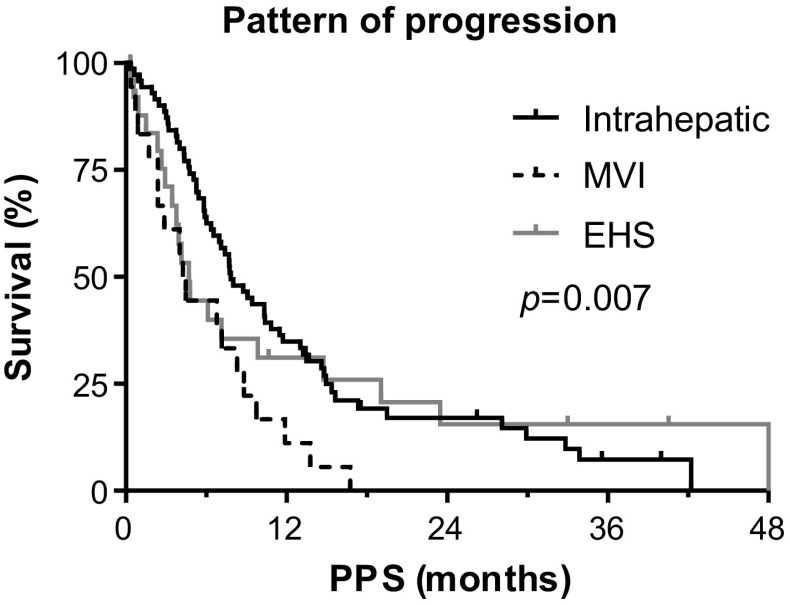
Post-progression survival (PPS) according to radiological pattern of progression. *EHS* extrahepatic spread; *MVI* macrovascular invasion

When comparing survival between different radiological patterns of progression, there was a poorer PPS in patients with MVI (4.2 months, 95% CI 3.3–5.2) or EHS (4.7 months, 95% CI 3.4–6.0) compared with patients with intrahepatic progression (10.3, 95% CI 7.8–12.9)(Fig. 4, log-rank *p* = 0.007). In a subgroup analysis in patients with preserved liver function and ECOG PS (*n* = 68), MVI remained an independent predictor of PPS (HR 1.74, 95% CI 1.74–2.85, *p *= 0.004) (Supplementary Table 3). The pattern of progression in this subgroup, including 42 (62%) patients with intrahepatic, 9 (13%) patients with MVI and 17 (25%) patients with EHS, was similar to the entire group of patients with radiological progression.

To estimate the impact of subsequent treatment on OS and PPS, we compared subgroups according to eligibility for subsequent treatment and receiving treatment or BSC only. Patients who were not eligible for subsequent treatment (*n* = 42) had the worst OS (15.9 months, 95% CI 6.7–25.1) and PPS (3.2 months, 95% CI 2.2–4.2). In patients eligible for subsequent treatment, receiving subsequent treatment was associated with a longer OS (21.7 vs 15.6, *p *= 0.103) and PPS (13.0 vs 4.8, *p *= 0.076) compared with BSC, although this was not statistically significant.

## Discussion

In this study of HCC patients treated with TACE, the majority of patients (58%) had preserved liver function and ECOG PS at UTP. These patients showed a trend toward better PPS when treated with subsequent liver-directed or systemic treatment compared with BSC only (13 vs 5 months). These results are in line with post hoc analyses of the landmark phase III sorafenib trials and a recent international observational study [27–29], showing a clear survival benefit in strictly selected patients treated with sorafenib after TACE compared with those receiving BSC only [29]. Although sorafenib is the guideline-recommended treatment strategy after TACE failure [1], the predominantly intrahepatic pattern of progression at UTP (75%) implies that most patients may be candidate for both liver-directed (radioembolization) and systemic treatments (sorafenib). Future studies might provide predictive markers that can guide the choice for radioembolization or sorafenib in order to maximize survival benefit of patients with UTP.

Although a minority of patients discontinued TACE because of hepatic decompensation (23%) or deteriorated ECOG PS (18%), these factors were independent factors for OS and PPS in our study. This underscores the need for assessment of liver function and ECOG PS after TACE treatment. The prognostic importance of these parameters in patients with HCC is widely accepted, reflected by its implementation in the BCLC staging system [1, 2]. Still, this is the first study to quantify its prognostic impact in context of switching from TACE to subsequent treatments. Prior studies reported 0–60% hepatic decompensation after TACE [3, 5, 6, 13, 30–32], depending on patient selection and the definition of hepatic decompensation. In our study, only 3 out of 27 (11%) patients who developed hepatic decompensation following TACE recovered enough to receive subsequent treatment. This may indicate that hepatic decompensation following is caused by a severe underlying liver disease or an aggressive tumor biology compromising liver function. These patients have a poor prognosis and are unlikely to benefit from the currently available subsequent treatments. In future studies, non-liver metabolized treatment options, i.e., immunotherapy, may be considered.

In concordance with a prior Japanese study [22], we showed that OS correlated strongly with TTUPc in HCC patients treated with TACE. This validates TTUPc as a novel surrogate endpoint for OS in both European and Japanese patients. Common surrogate endpoints in oncology such as time to progression (TTP) and progression-free survival (PFS) have limited accuracy in representing TACE success by not taking into account ‘reTACEable’ progression or the competing risk of underlying liver cirrhosis [15]. Therefore, current guidelines do not recommend TTP or PFS as endpoints in HCC [1]. TTUPc showed a strong correlation with OS and has the advantage of requiring a significantly shorter follow-up than OS (median of 13 vs 22 months), making it an interesting endpoint for future clinical trials in HCC investigating TACE or new treatments combined with TACE. Because patients are increasingly receiving multiple lines of anti-HCC treatment following TACE failure, this will result in a prolonged post-TACE survival (PPS) diluting the effect of TACE on OS. This highlights the value of TTUPc as a validated surrogate marker for TACE benefit.

Limitations to our study include the retrospective study design with its inherent drawbacks. Still, our study is representable for the multidisciplinary management of HCC patients undergoing TACE in a European country, with an OS that is similar to prior studies [26].

In conclusion, our data suggest that most patients discontinue TACE due to intrahepatic tumor progression with preserved ECOG PS and liver function, making them potential candidates for subsequent liver-directed or systemic treatment. Hepatic decompensation or deteriorated ECOG PS was independently associated with poor OS and PPS. TTUPc correlated strongly with OS, making this a potential surrogate endpoint for future trials estimating TACE benefit.

## Electronic supplementary material

Below is the link to the electronic supplementary material.

Supplementary material 1 (DOCX 23 kb)

Supplementary material 2 (JPEG 40 kb)

## Abbreviations

AFP: Alpha-fetoprotein
BCLC: Barcelona Clinic Liver Cancer
BSC: Best supportive care
CT: Computed tomography
cTACE: Conventional transarterial chemoembolization
DEB-TACE: Drug-eluting beads transarterial chemoembolization
EASL: European Association for the Study of the Liver
ECOG PS: Eastern Cooperative Oncology Group performance status
EHS: Extrahepatic spread
HBV: Hepatitis B virus
HCC: Hepatocellular carcinoma
HCV: Hepatitis C virus
IQR: Interquartile range
MDT: Multidisciplinary team
mRECIST: Modified response evaluation criteria in solid tumors
MRI: Magnetic resonance imaging
MVI: Macrovascular invasion
OS: Overall survival
PD: Progressive disease
PPS: Post-progression survival
RE: Radioembolization
RFA: Radiofrequency ablation
TACE: Transarterial chemoembolization
TTUPc: Time to untreatable progression by chemoembolization
UTP: unTACEable progression
