# Supplementary material for: Reason of Discontinuation After Transarterial Chemoembolization Influences Survival in Patients with Hepatocellular Carcinoma
Source: Cardiovasc Intervent Radiol. 2018 Nov 28;42(2):230–8. doi: 10.1007/s00270-018-2118-6 (PMC6344387; doi:10.1007/s00270-018-2118-6)
Supplement: Supplementary file 1 — Supplementary material 1 (DOCX 23 kb) [file 270_2018_2118_MOESM1_ESM.docx]

| **Supplementary Table 1.** Treatment details, adverse events of HCC patients treated with TACE | |
| --- | --- |
| **Variable** | **All patients (*N*=166)** |
| TACE details – n (%) |  |
| cTACE/deb-TACE | 33/133 (20/80) |
| Uni/Bilobar – n (%) | 128/38 (77/23) |
| Number of TACE (range) | 2 (1-7) |
| Received combined locoregional treatment – n (%) | 62 (37) |
| RFA/PEI | 58 (35) |
| Radioembolization | 3 (2) |
| SBRT | 1 (<1) |
| Acute adverse events (<24h) – n (%) |  |
| Post-embolic syndrome | 32 (19) |
| Bleeding puncture site | 3 (2) |
| Dissection/Bleeding | 2 (1) |
| Delirium | 3 (2) |
| Allergic reaction | 2 (1) |
| Delayed adverse events (<1 month) – n (%) |  |
| Liver dysfunction (Child-Pugh ≥B8) | 4 (2) |
| Prolonged/Severe post-embolic syndrome | 9 (5) |
| Liver abscess/Biloma | 3 (2) |
| Infection (other) | 5 (3) |
| Decompensatio cordis | 2 (1) |
| CVA | 1 (<1) |
| Death | 2 (1) |
| Liver dysfunction at TACE discontinuation – n (%) | 27 (16) |
| Yes, with recovery to Child-Pugh ≤B7 | 3 (2) |
| Yes, without recovery to Child-Pugh ≤B7 | 24 (15) |
| Abbreviations: cTACE, conventional transarterial chemoembolization; CVA, cerebrovascular accident; DEB-TACE, drug-eluting beads transarterial chemembolization; HCC, hepatocellular carcinoma; PEI, percutaneous ethanol injection; SBRT, stereotactic body radiotherapy (SBRT); cTACE, conventional transarterial chemoembolization; | |

| **Supplementary Table 2.** Univariable Cox regression analysis for overall survival. | | | | |
| --- | --- | --- | --- | --- |
|  | **Whole Cohort** | | **Patients with**  **unTACEable progression** | |
|  | n=166 | | n=116 | |
|  | **HR [CI 95%]** | **p-value*** | **HR [CI 95%]** | **p-value*** |
| **Prior to TACE-1** | | | | |
| Female sex | 1.05 (0.69-1.59) | 0.813 | 0.87 (0.55-1.39) | 0.558 |
| Age >68 | 0.87 (0.60-1.25) | 0.439 | 0.78 (0.51-1.17) | 0.223 |
| HBV | 0.87 (0.55-1.37) | 0.534 | 0.76 (0.44-1.31) | 0.321 |
| HCV | 0.97 (0.66-1.42) | 0.859 | 0.89 (0.57-1.40) | 0.890 |
| Alcohol | 1.05 (0.72-1.53) | 0.803 | 1.28 (0.84-1.96) | 0.248 |
| ECOG PS 2 (Ref: 0-1) | 2.38 (1.27-4.48) | **0.007** | 1.99 (0.95-4.17) | **0.068** |
| Child Pugh score B7 (Ref: A5-A6) | 1.54 (0.80-2.95) | 0.197 | 1.61 (0.77-3.36) | 0.202 |
| BCLC stage (Ref: 0/A)** | Ref | - | Ref | - |
| B | 1.99 (1.32-3.01) | **0.001** | 1.45 (0.93-2.27) | 0.106 |
| C | 4.43 (2.09-9.40) | **<0.001** | 3.62 (1.56-8.41) | **0.003** |
| Number of nodules (Ref: 1) | Ref | - | Ref | - |
| 2-3 | 1.55 (1.00-2.40) | **0.053** | 1.09 (0.65-1.81) | 0.752 |
| >3 / diffuse | 2.31 (1.44-3.72) | **0.001** | 1.92 (1.14-3.24) | **0.014** |
| Tumor size >46 mm | 1.57 (1.08-2.28) | **0.017** | 1.51 (1.01-2.27) | **0.044** |
| Macrovascular invasion | 2.91 (1.45-5.84) | **0.003** | 2.84 (1.30-6.22) | **0.009** |
| Log_10_ AFP | 1.37 (1.16-1.61) | **<0.001** | 1.43 (1.20-1.70) | **<0.001** |
| **At UnTACEable progression** | | | | |
| unTACEable progression | 2.42 (1.43-4.12) | **0.001** | NA (all progressors) | |
| Main reason (Ref: radiological PD) | - | - | Ref | - |
| Liver dysfunction | - | - | 1.74 (1.06-2.85) | **0.028** |
| ECOG PS >2 | - | - | 2.04 (1.18-3.54) | **0.011** |

*In bolt: *P*<0.1, included in multivariable model.
** Not included in multivariable analysis to avoid multicolinearity.

Abbreviations: AFP, Alpha-fetoprotein; BCLC, Barcelona Clinic Liver Cancer; ECOG PS, Eastern Cooperative Oncology Group Performance status; HBV, Hepatitis B virus; HCV, Hepatitis C virus, HCV; PD, progressive disease; TACE, transarterial chemoembolization;

| **Supplementary Table 3.** Univariable and multivariable Cox regression analysis for **PPS** in patients with UTP who are candidate for subsequent treatment (n=68) | | | | | |
| --- | --- | --- | --- | --- | --- |
|  | **Univariable** | | | **Multivariable** | |
|  | **HR [CI 95%]** | | ***p*-value*** | **HR [CI 95%]** | ***p*-value**** |
| **Prior to TACE-1** | | | | | |
| Female sex | 1.56 (0.81-2.99) | | 0.184 |  |  |
| Age >65 | 0.79 (0.46-1.38) | | 0.411 |  |  |
| HBV | 1.03 (0.48-2.20) | | 0.939 |  |  |
| HCV | 1.02 (0.56-1.87) | | 0.939 |  |  |
| Alcohol | 1.02 (0.53-1.96) | | 0.948 |  |  |
| ECOG PS 2 (Ref: 0-1) | 2.19 (0.52-9.21) | | 0.285 |  |  |
| Child Pugh score B7 (Ref: A5-A6) | 1.79 (0.64-5.05) | | 0.269 |  |  |
| BCLC stage (Ref: 0/A)*** | Ref | | - | - | - |
| B | 1.28 (0.72-2.28) | | 0.394 | - | - |
| C | 2.73 (0.91-8.19) | | **0.074** | - | **-** |
| Number of nodules (Ref: 1) | Ref | | - | Ref | - |
| 2-3 | 1.48 (0.76-2.87) | | 0.251 | 1.58 (0.75-3.34) | 0.232 |
| >3 / diffuse | 2.14 (1.03-4.42) | | **0.040** | 3.56 (1.61-7.90) | **0.002** |
| Tumor size >46 mm | 0.78 (0.45-1.36) | | 0.377 |  |  |
| Macrovascular invasion | 2.37 (0.83-6.75) | | 0.106 |  |  |
| Log_10_ AFP | 1.85 (0.97-3.54) | | **0.061** | 1.47 (1.12-1.92) | **0.005** |
| **At UnTACEable progression** | | | | | |
| Pattern of progression (Ref: Intrahepatic) | Ref | - | | Ref | - |
| MVI | 2.94 (1.38-6.29) | | **0.005** | 3.31 (1.45-7.53) | **0.004** |
| EHS | 0.97 (0.48-1.93) | | 0.919 | 1.19 (0.59-2.39) | 0.631 |

*In bolt: included in multivariable analysis (*p*-value <0.1)

**In bolt: *p*-value <0.05

*** Not included in multivariable analysis to avoid multicolinearity.

Abbreviations: AFP, Alpha-fetoprotein; BCLC, Barcelona Clinic Liver Cancer; ECOG PS, Eastern Cooperative Oncology Group Performance status; EHS, extrahepatic spread; HBV, Hepatitis B virus; HCV, Hepatitis C virus, HCV; MVI, macrovascular invasion; PD, progressive disease; TACE, transarterial chemoembolization;
