# Supplementary figures and images for: Reason of Discontinuation After Transarterial Chemoembolization Influences Survival in Patients with Hepatocellular Carcinoma
Source: Cardiovasc Intervent Radiol. 2018 Nov 28;42(2):230–8. doi: 10.1007/s00270-018-2118-6 (PMC6344387; doi:10.1007/s00270-018-2118-6)

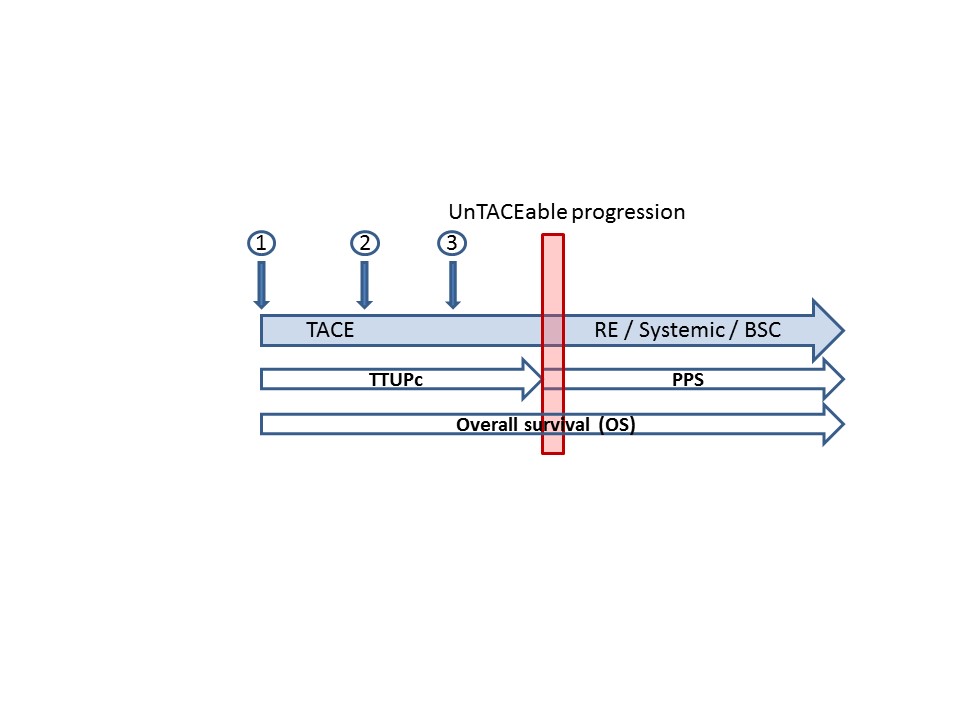

Supplement: Supplementary file 2 — Supplementary material 2 (JPEG 40 kb) [file 270_2018_2118_MOESM2_ESM.jpg]
